# Clinical Trial Landscape of Gene-Edited Autologous Hematopoietic Stem Cells for Hemoglobinopathies and Immunodeficiencies

**DOI:** 10.3390/ijms27083384

**Published:** 2026-04-09

**Authors:** Karen O’Hanlon Cohrt, Shirley O’Dea

**Affiliations:** 1 CRISPR Medicine ApS, Kong Georgs Vej 12, 2000 Frederiksberg, Denmark; karenarabellaohanlon@gmail.com; 2 Biology Department, Maynooth University, W23 F2H6 Kildare, Ireland

**Keywords:** autologous stem cell therapy, hematopoietic stem and progenitor cells, CRISPR/Cas9, base editors, zinc finger nucleases, gene editing, sickle cell disease, β-thalassemia, clinical trials, bench-to-bedside translation

## Abstract

Allogeneic hematopoietic cell transplantation (HCT) has been used for decades to treat certain malignant and non-malignant hematological conditions, but challenges remain. Increased understanding of disease mechanisms and recent developments in genome editing have enabled alternative strategies utilizing gene-edited autologous HCT and many of these have progressed to the clinic. We present here a comprehensive review of clinical trials of gene-edited autologous hematopoietic stem cells for the treatment of hemoglobinopathies and immunodeficiencies. Searches of major international clinical trial registries were carried out using specific key words. In total, 44 interventional clinical trials investigating gene-edited autologous stem cell therapies were identified, with CASGEVY (exagamglogene autotemcel) being the only product approved to date. Hemoglobinopathies were the most common indication (*n* = 37) followed by immunodeficiencies (*n* = 4), with single trials in HIV-1 infection, pyruvate kinase deficiency and limb–girdle muscular dystrophy. Gene-editing strategies fall into three categories: disruption of the BCL11A erythroid enhancer, editing of the γ-globin promoter and direct correction or disruption of disease-relevant genes. CD34^+^ hematopoietic stem and progenitor cells are the most common cell types edited, and CRISPR-Cas9 is the most widely used gene-editing modality. While results are encouraging, efficient intracellular delivery of gene-editing tools, editing efficiencies and off-target editing remain challenges for the field.

## 1. Introduction

The hematopoietic system comprises all blood-forming tissues, including the bone marrow, lymph nodes, spleen, liver and thymus, as well as a range of circulating specialized blood cell types, including erythrocytes, megakaryocytes and adaptive and innate immune cells. Hematopoietic stem cells (HSCs) are essential for replenishing blood cells, a process known as hematopoiesis. Capable of both self-renewal and multipotent differentiation, HSCs are primarily found in the bone marrow in adults, with a small number also circulating in the peripheral blood. Umbilical cord blood is also a rich source. The term hematopoietic stem and progenitor cells (HSPCs) includes both HSCs and progenitor cells which have become committed to differentiating into certain lineages and lack self-renewal ability [1]. CD34 is expressed on most HSPCs and the terms HSC, HSPC and CD34^+^ cells tend to be used interchangeably, despite being distinct.

The ability of HSCs to reconstitute the entire blood system enables hematopoietic cell transplant (HCT), a procedure whereby stem cells are infused into patients with various cancers or certain non-malignant conditions following a short course of chemotherapy or radiotherapy [2]. Most HCTs are carried out to cure an underlying malignancy or hematologic disorder. HSCs can be obtained from the recipient (autologous) or from donors (allogeneic) from bone marrow, peripheral blood or cord blood. Both autologous and allogeneic HCT are performed to allow the recipient to recover from a high-dose chemotherapeutic conditioning regimen, with or without radiotherapy. In addition, with allogeneic HCT, the immune cells in the graft may provide a graft-versus-tumor response which may be beneficial for certain patients. While, overall, the number of autologous transplants exceeds that of allogeneic transplants [3], the decision between autologous and allogeneic transplantation is guided by which approach has demonstrated superior efficacy in clinical studies [2]. Worldwide, more than 50,000 HCT procedures are performed annually with most performed for hematologic malignancies [4]. Non-malignant disorders suitable for HCT include autoimmune diseases such as multiple sclerosis, systemic sclerosis and certain cases of Crohn’s disease and systemic lupus erythematosus among others, as well as inherited diseases including severe combined immunodeficiency (SCID), thalassemia and sickle cell disease (SCD) [4].

**Figure 1:**
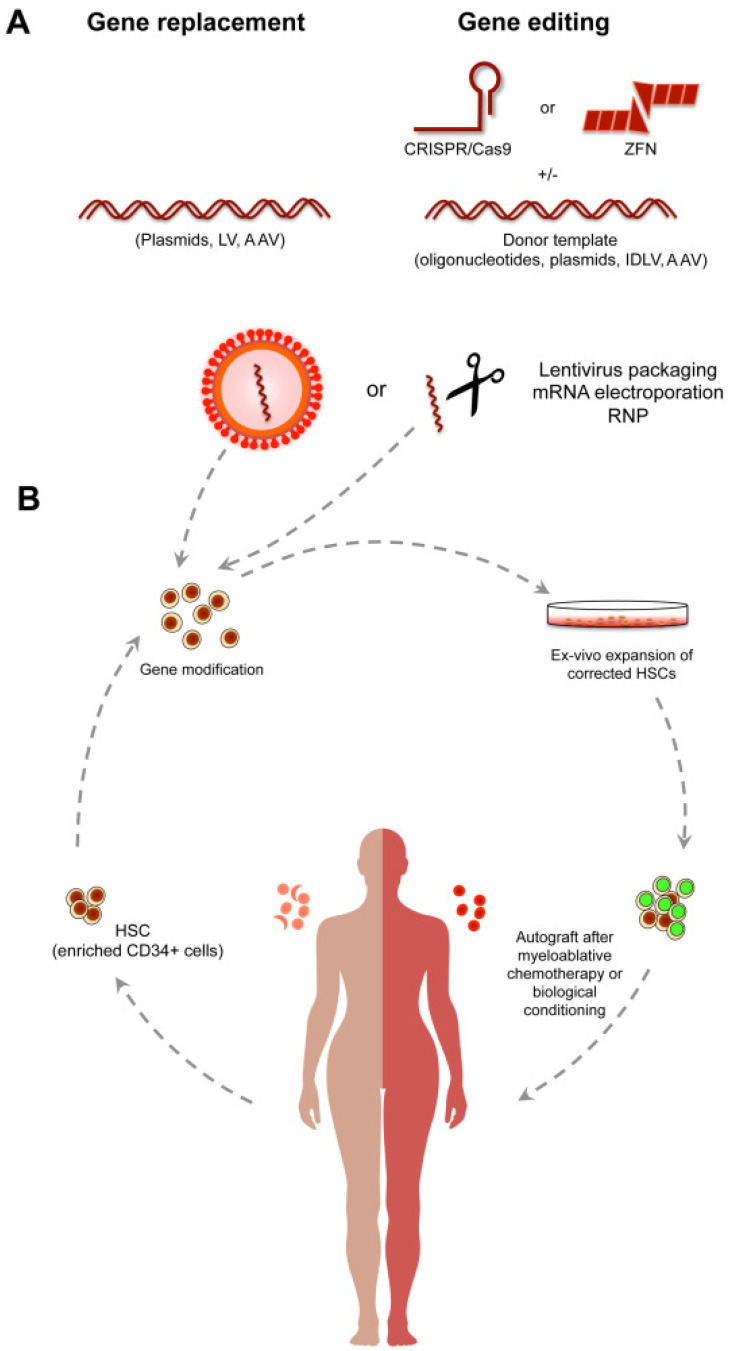
Strategies for gene replacement and gene-editing therapy for hemoglobinopathies. (**A**). Molecular tools used in gene therapy by gene replacement and by gene editing. (**B**). Scheme representing the stepwise procedure of gene therapy: autologous transplantation of cells genetically modified by gene replacement or gene editing in patients affected by β-thalassemia or sickle cell disease. Red cells represent unmodified cells and green cell represent gene-modified cells. Nucleases are represented by a scissor. LV: lentiviral vector; AAV: adeno-associated vector; ZFN: zinc finger nuclease; IDLV: integrase defective lentiviral vector; RNP: ribonucleoprotein. Figure and modified legend are reproduced with permission from Cold Spring Harbor Laboratory Press [7].

While allogeneic HCT has been used for decades as a curative treatment for certain non-malignant conditions, significant challenges exist include sourcing of suitable donors as well as risks of graft-versus-host disease, graft failure and treatment-related mortality [5]. Recent discoveries and developments in the field of gene editing have meant that allogeneic HSCs can be modified to improve functionality [6]. However, to date, gene therapy/editing has been explored more extensively for autologous HCT towards curative treatment for several inherited blood disorders (Figure 1) [7]. Gene therapy by gene addition, involving the semi-random integration of one or more copies of a therapeutic gene into the HSC genome, has long been studied for inherited disorders [8]. Retroviruses are typically used as vectors due to their ability to stably transduce HSCs. Serious adverse events during early studies led to the development of safer gamma-retroviral and lentiviral vectors but the risk of insertional mutagenesis remains [8]. Progress in gene-editing technologies has stimulated studies exploring the ability of these tools to correct mutations in HSCs. Here we present a comprehensive overview of clinical trials for hemoglobinopathies and immunodeficiencies that utilize gene-edited autologous HSCs. The development and approval of CASGEVY is used as a central case study to illustrate bench-to-bedside translation.

## 2. Gene-Editing Tools

In recent years, remarkable progress in genetic engineering, and gene editing in particular, has been achieved. The following is a brief overview of the gene-editing tools used in the clinical trials covered herein.

### 2.1. Zinc-Finger Nucleases

Zinc-finger nucleases (ZFNs) are engineered proteins consisting of a DNA-binding domain fused to the cleavage domain of the FokI nuclease and were the first programmable nucleases developed to enable targeted genome editing in mammalian cells [9]. The DNA-binding domain comprises an array of zinc-finger motifs, each of which typically recognizes a specific 3-nucleotide sequence triplet of DNA bases. Two ZFN monomers bind to adjacent DNA sequences to induce a double-stranded break (DSB), following which a DNA repair process called non-homologous end joining (NHEJ) is initiated by the cell. This process is error prone, often resulting in insertions or deletions of nucleotides (indels), which may lead to a reduction in gene expression, thus achieving functional gene knockout. In a related process, insertion of a DNA sequence, or knockin, can be achieved. For this, the desired DNA segment is co-delivered with the ZFN and can be inserted into the cut site in a process called homology-directed repair (HDR) (Figure 1) [7]. However, ZFN-mediated gene editing has been limited by the requirement for extensive protein engineering expertise as well as off-target cleavage events and cytotoxicity.

### 2.2. CRISPR-Cas9 and CRISPR-Cas12 Nucleases

First discovered in bacteria as a defense mechanism against viral infection, clustered regularly interspaced short palindromic repeats (CRISPRs)-associated (Cas) proteins are endonucleases that catalyze site-specific cleavage of double-stranded DNA [9]. Several Cas proteins have now been identified in various bacterial systems and Cas9, followed by Cas12a, are the most widely used as gene-editing tools. These proteins combine with a short guide RNA (gRNA) molecule to form a ribonucleoprotein (RNP) (Figure 1). The gRNAs are complementary to the target DNA sequence which is known as the protospacer. For gene editing, the RNP enters the nucleus and the Cas9 protein scans the DNA for a short recognition sequence called protospacer adjacent motif (PAM) which must be immediately downstream of the target DNA sequence. If the adjacent DNA sequence matches the gRNA, Cas9 creates a DSB in the DNA at that site. Gene knockout or knockin can be achieved via NHEJ or HDR respectively. Differences between Cas9 and Cas12 include the employment of different PAM sites and the production of blunt cuts by Cas9 compared with staggered cuts by Cas12a, the latter of which are beneficial for gene insertion. However, limitations of CRISPR-Cas systems include unintended and unpredictable genome alterations including off-target indels, large-scale deletions at on-target sites, chromosome losses or truncations and potential genotoxicity of DSBs [10,11].

### 2.3. Base Editors

To address the limitations associated with CRISPR-Cas systems, base and prime editors have been developed to enable precise single or multiple base substitutions in DNA without introducing DSBs. Base editors (BEs) are fusion proteins comprising a modified Cas9 nickase (nCas9) fused to a nucleotide deaminase enzyme [12]. In these systems, the gRNA guides the complex to the target site in the genome, the Cas9 nickase induces a single strand break and the specific deaminases catalyze deamination of the single-stranded DNA. While cytosine base editors (CBEs) and adenine base editors (ABEs) were first developed, mediating the conversion of cytosine (C)-to-thymine (T) and adenine (A)-to-guanine (G) respectively, a number of BEs have now been designed that can achieve a range of base edits [12]. While avoiding the issue of DSBs seen with CRISPR-Cas systems, off-target base editing can still occur, and this is believed to be primarily caused by the deaminases.

### 2.4. Glycosylase Base Editors

To further improve gene-editing efficiency and specificity, deaminase-free BEs have been developed which contain a glycosylase fused to a nCas9 to directly perform base editing without deamination [13]. It is suggested that these systems may have advantages over deaminase-based BEs due to their smaller size, simpler cellular mechanism and wider base-editing types; however, off-target edits persist [14].

### 2.5. Prime Editors

Prime-editing systems were developed to improve precision and flexibility of gene editing. These systems comprise nCas9 with the nuclease domain deactivated, an engineered reverse transcriptase (RT) domain, and a prime-editing guide RNA (pegRNA) that incorporates a reverse transcription template (RTT) and an additional primer binding sequence [10]. The nCas9 generates a single strand break at the target site in the genomic DNA and the RT reads the RNA template built into the pegRNA to synthesize a complementary DNA strand. This newly made DNA fragment containing the desired edit is then incorporated into the genome during DNA repair. Low editing efficiency and off-target edits remain a challenge with these systems [10].

## 3. Clinical Trial Identification and Data Sources

We identified 44 interventional clinical trials investigating gene-edited autologous stem cell therapeutic candidates and these are summarized in Table 1 and Table 2. The trials were identified primarily through the Clinical Trials Register curated by CRISPR Medicine News (CMN) (https://crisprmedicinenews.com/trialdb/, URL last accessed on 31 December 2025). This register contains publicly disclosed gene-editing clinical programs. To maximize coverage, CMN’s resource was complemented by systematic searches of major international clinical trial registries, including ClinicalTrials.gov (National Institutes of Health [NIH], Bethesda, MD, USA), the Chinese Clinical Trial Registry (ChiCTR), the EU Clinical Trials Register, the ISRCTN registry (United Kingdom), and the Australia and New Zealand Clinical Trials Registry (ANZCTR).

We performed searches at the aforementioned international registries using various combinations of keywords related to gene-editing technologies (including “gene editing”, “CRISPR”, “Cas9”, “Cas12”, “base editing”, “prime editing”, “zinc finger nuclease”, “TALEN”, and “meganuclease”) together with terms describing autologous stem or progenitor cell therapies (including “autologous”, “stem cell”, “hematopoietic stem cell”, “HSPC”, “CD34^+^”, and “genetically modified”).

Identified trials were manually curated to confirm the use of genome-editing technologies, and that any candidate therapy under investigation was patient-derived and of stem or progenitor cell source. The final search was completed on 31 December 2025. While we cannot rule out the possibility that additional studies exist that are not publicly registered or disclosed, we believe that the trials presented herein provide a comprehensive and representative overview of clinical-stage gene-edited autologous stem cell therapies worldwide at the time of analysis.

## 4. Broad Overview of All Clinical Trials Uncovered in This Review

The 44 identified trials are summarized in Table 2 and reflect a rapidly growing and increasingly diversified clinical pipeline. The vast majority of studies focus on hemoglobinopathies (*n* = 37), inherited disorders of hemoglobin synthesis, more specifically sickle cell disease (SCD) and β-thalassemia, which together represent the most clinically advanced applications of autologous stem cell editing. A smaller subset of trials target primary immunodeficiencies (*n* = 4), including X-linked severe combined immunodeficiency (X-SCID), X-linked chronic granulomatous disease (X-CGD), autosomal recessive chronic granulomatous disease, and CD40L-linked Hyper-IgM syndrome. The remaining trials (*n* = 3) explore edited autologous stem cells in other disease contexts, including single trials in HIV-1 infection, the inherited metabolic disorder pyruvate kinase deficiency (PKD) and limb–girdle muscular dystrophy (LGMD), the latter involving gene-edited muscle satellite stem cells rather than hematopoietic progenitors. Collectively, these 44 trials illustrate the dominance of hematological indications in the field and the emergence of gene-edited autologous stem cell therapies in other disease areas.

Most of the identified trials are in the early phase, with Phase 1 or combined Phase 1/2 studies comprising the majority (*n* = 34), reflecting an overall landscape that is still maturing clinically. Despite the early-phase dominance referred to above, a considerable number of trials (*n* = 6) have progressed to late-stage evaluation, including several Phase 3 trials of CRISPR-Cas9-edited CD34^+^ HSPCs for sickle cell disease (SCD) and β-thalassemia (BT). Importantly, these clinical efforts have culminated in the first regulatory approval of a gene-edited autologous stem cell therapy, exagamglogene autotemcel (exa-cel; marketed as CASGEVY). Exa-cel is a CRISPR-Cas9-edited autologous stem cell therapy developed for the treatment of SCD and transfusion-dependent β-thalassemia (TDT) through targeting of the *BCL11A* erythroid enhancer, and it remains the only gene-edited therapy approved to date.

Although recruitment occurs across multiple countries, most trials are sponsored by organizations based in the United States or China. A small number of trials are led by sponsors based in other regions, most notably Europe (e.g., NCT05588401). Across the 44 trials, CRISPR-based technologies (including those based on Cas9, Cas12a, and Cas12b) dominate (*n* = 28), while base editing (*n* = 12), ZFNs (*n* = 3) and prime editing (*n* = 1) are also represented, highlighting the increasing diversification of gene-editing platforms entering the clinic.

While the approaches described above rely primarily on ex vivo editing of autologous hematopoietic stem and progenitor cells followed by myeloablative conditioning, several emerging strategies may influence the next generation of gene-editing therapies. These include the development of chemotherapy-free conditioning approaches, in vivo genome-editing strategies designed to target hematopoietic stem cells directly within the body, and new delivery platforms such as lipid nanoparticles (LNPs) and virus-like particles (VLPs) [32,33,34]. These developments are currently advancing largely within preclinical programs but have the potential to improve the safety, accessibility, and scalability of gene-editing therapies and may influence the design of future clinical trials.

## 5. Gene-Editing Approaches to Treat Hemoglobinopathies

As outlined above, hemoglobinopathies account for the largest proportion of current clinical trials of gene-edited autologous stem cell therapies. SCD and TDT arise from mutations in the hemoglobin β-subunit gene, *HBB*, which encodes a critical component of the major oxygen-carrier adult hemoglobin A (HbA). Both diseases belong to the broader group of inherited hemoglobin disorders collectively termed hemoglobinopathies. SCD and TDT are among the most common monogenic diseases worldwide, with approximately 60,000 and 300,000 new diagnoses reported annually, respectively [19].

## 6. Etiology of Hemoglobinopathies and Current Treatments

TDT can arise through more than 200 known mutations in *HBB*, resulting in severely reduced or absent β-globin synthesis and an imbalance between the α-like and β-like globin chains that make up HbA [35]. This leads to insufficient mature, functional adult hemoglobin and ineffective erythropoiesis, chronic anemia, dysregulated iron metabolism and organ toxicity, all of which contribute to poorer quality of life, reduced life expectancy and increased morbidity [35,36,37,38,39,40].

SCD arises through a single point mutation in *HBB* that substitutes glutamic acid with valine at amino acid position 6. This results in the production of sickle hemoglobin (HbS) which polymerizes to form long, rigid chains when oxygen levels are low. This causes red blood cells to become crescent- or sickle-shaped and compromises their ability to pass through small blood vessels. SCD manifests as a complicated disease involving red blood cell deformation, hemolysis, anemia, painful vaso-occulative episodes caused by the inability of sickled cells to pass through small blood vessels, irreversible organ damage and reduced life expectancy [19,41].

Standard treatment approaches to SCD and TDT are non-curative, including blood transfusions, pain management and symptom relief. Additionally, iron chelation therapy is used to manage iron overload in TDT patients receiving blood transfusions and the anti-cancer agent hydroxyurea is used in the treatment of SCD [42,43,44]. Hydroxyurea was the first therapy to receive FDA approval for SCD (in 1997). It promotes the production of fetal hemoglobin and is widely used in the treatment of SCD today, although its mechanism of action remains poorly defined [43,44].

Recently approved therapies including luspatercept (a recombinant protein-based therapy that promotes erythroid maturation) and crizanlizumab (a monoclonal antibody that improves the flow of sickled red blood cells through blood vessels) have reduced some symptoms of TDT and SCD, respectively, but neither addresses the root cause of disease nor fully ameliorates all disease manifestations [45,46]. Allogeneic bone marrow transplantation has curative potential for TDT and SCD, but the severe shortage of suitable donors and life-threatening complications associated with transplantation renders this approach unrealistic for the vast majority of patients [36,47,48].

Two gene-addition therapies have gained regulatory approval for hemoglobinopathies. These include Zynteglo^®^ (betibeglogene autotemcel), a lentiviral vector-based β-globin gene-addition therapy approved for TDT, and Lyfgenia^®^ (lovotibeglogene autotemcel), a similar lentiviral gene-addition therapy approved for the treatment of SCD in the United States, both of which rely on lentiviral gene addition rather than targeted genome modification [49,50,51].

## 7. Breakthrough Discoveries in Globin Gene Regulation That Enabled New Therapeutic Strategies for Hemoglobinopathies

**Figure 2:**
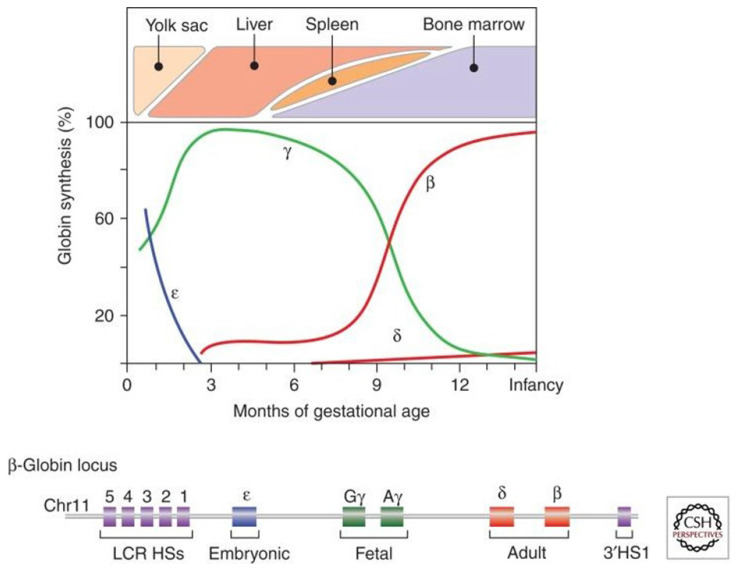
The fetal-to-adult hemoglobin switch. This illustration depicts the normal timing of the developmental hemoglobin switches in humans. In the (**top**) panel, the sites and levels of various β-like globin molecules are shown with colors corresponding to the various developmental groups of genes shown below in a model of the human β-globin locus (embryonic in blue, fetal in green, and adult in red). The (**bottom)** illustration also depicts the upstream enhancer of the β-globin locus, known as the locus control region (LCR), with its corresponding DNAse I hypersensitivity sites (HSs) and a downstream HS known as the 3′HS1. Figure and legend are reproduced with permission from Cold Spring Harbor Laboratory Press [59].

A major advance in the treatment of hemoglobinopathies arose from clinical observations that persistence of fetal hemoglobin (HbF) expression into adulthood markedly ameliorates disease severity in both SCD and TDT [41,52,53,54,55,56,57,58]. Under normal physiological conditions, HbF expression is developmentally silenced shortly after birth through a tightly regulated transcriptional switch from γ-globin to β-globin production, such that adult red blood cells contain predominantly HbA [59], as shown in Figure 2.

Individuals with hereditary persistence of fetal hemoglobin (HPFH) exhibit reduced clinical manifestations of SCD or TDT despite carrying pathogenic *HBB* mutations, demonstrating that elevated HbF can functionally compensate for defective adult β-globin [52]. These observations established HbF reactivation as a compelling therapeutic strategy that could treat both SCD and TDT through modulation of globin gene regulation rather than direct correction of disease-causing *HBB* mutations. Consistent with this rationale, the majority of gene-editing strategies currently in clinical development for hemoglobinopathies center on reactivation of fetal hemoglobin through modification of globin regulatory elements, rather than direct correction of disease-causing *HBB* mutations (see Table 1 and Table 2).

Decades of molecular and genetic studies (detailed in [59]) carried out to understand the regulation of fetal hemoglobin expression culminated in the identification of three genomic loci harboring common variants associated with inter-individual differences in HbF levels [60,61,62,63]. Among these, the B-cell lymphoma/leukemia 11A (*BCL11A*) gene on chromosome 2 emerged as a central, developmentally regulated repressor of γ-globin expression [64,65]. *BCL11A* encodes a zinc-finger transcription factor, and functional studies demonstrated that genetic inactivation or knockdown of *BCL11A* robustly induces HbF production in adult erythroid cells without significantly impairing erythroid differentiation, establishing BCL11A as a target for therapeutic HbF reactivation [65,66,67,68].

Despite its strong therapeutic rationale, direct targeting of *BCL11A* initially posed a major translational challenge, as loss of BCL11A function outside the erythroid lineage is incompatible with normal hematopoiesis, particularly B-lymphocyte development, thereby limiting the feasibility of global BCL11A inhibition [62]. Subsequent mechanistic studies identified an erythroid-specific enhancer element controlling *BCL11A* expression, revealing a strategy by which BCL11A activity could be selectively attenuated in erythroid cells [69]. This discovery provided the foundation for current therapeutic approaches that target the erythroid-specific *BCL11A* enhancer to induce fetal hemoglobin while preserving essential BCL11A functions in other hematopoietic lineages (see Table 1).

**Scheme 1:**
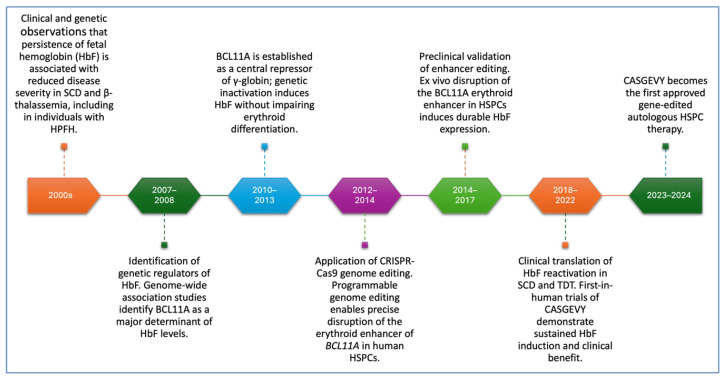
Development timeline of CASGEVY (exagamglogene autotemcel).

The emergence of genome-editing technologies enabled direct, loss-of-function interrogation of regulatory DNA elements controlling the fetal-to-adult globin switch, exemplified by targeted disruption of the erythroid-specific *BCL11A* enhancer [70,71]. In the case of CASGEVY, CRISPR-Cas9-mediated disruption of the erythroid-specific enhancer of *BCL11A* selectively reduces *BCL11A* expression in erythroid cells, resulting in durable fetal hemoglobin induction with an HPFH-like phenotype that enabled the first approved gene-edited autologous stem cell therapy (see Scheme 1). In parallel, alternative HbF induction strategies are advancing clinically that more directly mimic naturally occurring HPFH mutations, including editing of the γ-globin gene (i.e., *HBG1*/*HBG2*) promoters to disrupt repressor binding sites (e.g., for BCL11A and others) to maintain HbF expression into adulthood (see Table 1 and Table 2).

## 8. Clinical Data Supporting Regulatory Approval of CASGEVY

Phase 3 clinical data for CASGEVY were published in 2024 from pivotal clinical trials in SCD and TDT [20,50,72]. In both trials, patients received a single infusion of CASGEVY following myeloablative busulfan conditioning, which was required to facilitate durable engraftment of edited cells.

In SCD, efficacy was evaluated in the single-group, open-label CLIMB SCD-121 study (NCT03745287). This study enrolled 44 adolescents and adults (12 to 35 years old) with SCD who had suffered at least two severe VOCs in each of the two years before screening. The primary and key secondary endpoints were freedom from severe VOCs for a continuous period of at least 12 months and freedom from hospitalization for severe VOCs for 12 consecutive months. Of the patients eligible for analysis, 97% and 100% met the primary and key secondary endpoints, respectively. Patients exhibited early and sustained increases in total hemoglobin and HbF levels, and improvements were seen in all markers of hemolysis evaluated [72].

In TDT, Phase 3 data were obtained from the CLIMB THAL-111 study (NCT03655678), which enrolled 52 adolescents and adults (12 to 35 years old) with TDT who had a high burden of red blood cell transfusions and impaired life quality. The primary endpoint was transfusion independence for at least 12 consecutive months while maintaining adequate hemoglobin levels. Of all enrolled patients, 97% achieved transfusion independence, with hemoglobin levels maintained predominantly through durable HbF induction [20].

In both trials, durable engraftment of edited CD34^+^ cells and sustained HbF expression were observed over reported follow-up periods extending beyond one year. The safety profile of CASGEVY was consistent with that expected following myeloablative conditioning and autologous hematopoietic cell transplantation, with no serious adverse events attributed to gene editing and no evidence of clonal dominance or malignant transformation during follow-up [20,50,72]. Together, these Phase 3 studies represent the most advanced clinical data reported to date for a gene-editing therapeutic.

## 9. Global Regulatory Approval of CASGEVY

Exagamglogene autotemcel (Exa-cel; marketed as CASGEVY), developed by Vertex Pharmaceuticals and CRISPR Therapeutics, was first approved by the UK Medicines and Healthcare products Regulatory Agency in November 2023 for the treatment of both sickle cell disease and transfusion-dependent β-thalassemia. This was followed by breakthrough approval from the US Food and Drug Administration in December 2023 for sickle cell disease and subsequently in January 2024 for transfusion-dependent β-thalassemia. Conditional approval was granted by the European Commission in February 2024 for both indications. Beyond the UK, United States, and European Union, CASGEVY has received regulatory approval in several additional jurisdictions, including Canada, Switzerland, Saudi Arabia, and Bahrain in 2024, with further approvals following in the United Arab Emirates and Qatar in 2025.

## 10. Gene-Editing Approaches to Treat Immunodeficiencies

In addition to hemoglobinopathies, a growing number of clinical trials are evaluating gene-edited autologous hematopoietic stem and progenitor cell (HSPC) therapies for the treatment of inherited immunodeficiencies (*n* = 4; Table 2). These studies focus on monogenic disorders caused by loss-of-function mutations in genes that are essential for immune cell development or effector function and predominantly employ direct gene correction or inactivation strategies (see targeting rationales in Table 1).

Current clinical programs include three Phase 1/2 trials sponsored by the National Institute of Allergy and Infectious Diseases (NIAID), targeting distinct primary immunodeficiencies through base editing of autologous HSPCs. These trials, which are either recruiting or enrolling by invitation, involve correction of *IL2RG* mutations in X-linked severe combined immunodeficiency (X-SCID; NCT06851767), correction of *CYBB* mutations in X-linked chronic granulomatous disease (X-CGD; NCT06325709), and correction of *CD40LG* mutations in CD40L-associated Hyper-IgM syndrome (NCT06959771) [73,74]. An ongoing Phase 1/2 trial sponsored by Prime Medicine is evaluating PM359, a prime-edited autologous HSPC therapy designed to correct pathogenic mutations in *NCF1* for the treatment of autosomal recessive p47^phox-deficient chronic granulomatous disease (NCT06559176). Initial clinical data published in late 2025 demonstrated rapid hematopoietic engraftment, durable restoration of NADPH oxidase activity, and early clinical benefit without genome-editing-related safety concerns, providing the first clinical validation of prime editing in autologous HSPCs [31].

## 11. Clinical-Stage Gene-Editing Approaches Beyond Hematologic Indications

A small number of additional trials extend gene-editing approaches beyond classical hemoglobinopathies and primary immunodeficiencies (*n* = 3; Table 2). A Phase 1 trial in HIV-1 infection, sponsored by Sangamo Therapeutics, is evaluating ZFN-mediated disruption of the host *CCR5* co-receptor in autologous CD34^+^ HSPCs to reduce viral susceptibility, representing a host-directed therapeutic strategy rather than correction of a disease-causing mutation (NCT02500849) [75]. In addition, a recruiting Phase I study in pyruvate kinase deficiency (PKD), sponsored by Shanghai Children’s Medical Center, is assessing CRISPR/adeno-associated virus (AAV6)-mediated targeted insertion of a codon-optimized *PKLR* cDNA into autologous CD34^+^ HSPCs to restore pyruvate kinase activity and erythroid metabolic function (ChiCTR2300073795). In contrast to the hematopoietic focus of most of the trials discussed herein, a single trial sponsored by Simone Spuler, MD, at Charite University in Berlin applies CRISPR-Cas9 editing to an undisclosed genomic target in autologous muscle satellite stem cells for LGMD (NCT05588401) [76], highlighting the application of genome-editing technologies into non-hematopoietic stem cell compartments.

## 12. Conclusions

Advances in understanding of disease genetics and the ongoing development of genome-editing tools are informing strategies for novel approaches to treating blood disorders. The approval of CASGEVY represents a pivotal milestone, validating the clinical feasibility of personalized, gene-edited cell therapies. However, the high cost and complex manufacturing associated with such therapies present important challenges for global accessibility, prompting initiatives such as the Global Gene Therapy Initiative aimed at improving equitable access to these treatments worldwide [77]. Many other approaches have moved into the clinic and results will inform future strategies. Technical challenges with gene-editing tools continue to be discovered and addressed, driving iterative improvements in editing precision and safety that will be critical for the broader and more durable application of these therapies. Emerging computational approaches and advances in conditioning strategies, in vivo genome-editing methods, and next-generation delivery platforms such as LNPs and VLPs may further support the evolution of gene-editing therapies and shape the design of future clinical trials.

## Figures and Tables

**Table 1:** Mechanism of action for gene-editing strategies used in autologous HSPC-based therapies.

Strategy No.	Strategy	Mechanism of Action
1	*BCL11A* erythroid enhancer disruption	Disruption of the erythroid-specific enhancer of *BCL11A* to derepress γ-globin expression and induce fetal hemoglobin, compensating for defective β-globin in sickle cell disease and β-thalassemia.
2	γ-globin promoter editing (to mimic HPFH)	Editing of *HBG1*/*HBG2* promoter or repressor-binding motifs to recapitulate naturally occurring HPFH variants and maintain fetal hemoglobin expression into adulthood.
3	Direct correction or inactivation of disease-relevant genes	Direct correction or inactivation of disease-relevant genes at the endogenous locus to restore normal or therapeutically beneficial cellular function, including globin genes (*HBB*, *HBA2*), genes implicated in inherited immunodeficiencies (e.g., *IL2RG*, *CYBB*, *NCF1*, *CD40LG*), and host susceptibility factors (e.g., *CCR5*) targeted as a therapeutic strategy to prevent HIV-1 entry.

Abbreviations: *BCL11A*: B-cell lymphoma/leukemia 11A; *CCR5*: CC chemokine receptor type 5; *CD40LG*: CD40 ligand; *CYBB*: Cytochrome b-245 beta chain; *HBA2*: Hemoglobin subunit alpha 2; *HBB*: Hemoglobin subunit beta; *HBG1*: Hemoglobin subunit gamma 1; *HBG2*: Hemoglobin subunit gamma 2; HIV-1: Human immunodeficiency virus type 1; HPFH: Hereditary persistence of fetal hemoglobin; *IL2RG*: Interleukin-2 receptor subunit gamma; *NCF1*: Neutrophil cytosol factor 1 (also known as p47phox).

**Table 2:** Global clinical trials of gene-edited autologous stem cell therapies (note: we have retained the cell type nomenclature that was reported in the source clinical trials databases).

Disease	Trial Identifier	Phase	Candidate	Target Gene	MOA	Age (yrs)	Sponsor	Location(s)	Cell Type	Gene Editor	Status	Start Date	Trial Data Available
SCD	NCT03653247	1/2	BIVV003	BCL11A erythroid enhancer	1	18 to 40	Sangamo Therapeutics	US	CD34^+^ HSCs	ZFN	Completed	6 March 2019	[15]
BT	NCT03432364	1/2	ST-400	BCL11A erythroid enhancer	1	18 to 40	Sangamo Therapeutics	US	CD34^+^ stem cells	ZFN	Completed	29 March 2018	[16]
SCD	NCT06300723	NA	BRL-101	BCL11A erythroid enhancer	1	3 to 35	BRL Medicine	China	CD34^+^ HSPCs	CRISPR-Cas9	Enrolling by invitation	29 July 2024	[17]
SCD	NCT06287086	NA	BRL-101	BCL11A erythroid enhancer	1	3 to 35	BRL Medicine	n.d.	CD34^+^ HSPCs	CRISPR-Cas9	Not yet recruiting	14 June 2024	No
SCD	NCT06287099	NA	BRL-101	BCL11A erythroid enhancer	1	3 to 35	BRL Medicine	n.d.	CD34^+^ HSPCs	CRISPR-Cas9	Not yet recruiting	20 April 2024	No
BT	NCT04205435	1/2	BRL-101	BCL11A erythroid enhancer	1	5 to 15	BRL Medicine	China	CD34^+^ HSPCs	CRISPR-Cas9	Terminated	1 November 2021	[18]
BT	NCT04211480	NA	BRL-101	BCL11A erythroid enhancer	1	5 to 15	BRL Medicine	China	CD34^+^ HSPCs	CRISPR-Cas9	Completed	1 October 2020	[18]
BT	NCT05577312	1	BRL-101	BCL11A erythroid enhancer	1	3 to 35	BRL Medicine	China	CD34^+^ HSPCs	CRISPR-Cas9	Enrolling by invitation	1 November 2022	[18]
BT	NCT04390971	NA	ET-01	BCL11A erythroid enhancer	1	6 to 35	IHBDS Hospital, China	China	CD34^+^ HSPCs	CRISPR-Cas9	Unknown	10 February 2023	No
BT	NCT04925206	1	ET-01	BCL11A erythroid enhancer	1	12 to 35	EdiGene	China	CD34^+^ HSPCs	CRISPR-Cas9	Terminated	17 August 2021	No
BT	NCT05752123	NA	ET-01	BCL11A erythroid enhancer	1	6 to 35	EdiGene	China	CD34^+^ HSPCs	CRISPR-Cas9	Terminated	18 February 2023	No
BT	NCT03655678	2/3	Exa-cel (CASGEVY)	BCL11A erythroid enhancer	1	12 to 35	Vertex Pharmaceuticals	US, Canada, Germany, Italy, UK	CD34^+^ HSPCs	CRISPR-Cas9	Completed	14 September 2018	[19,20]
SCD	NCT03745287	1/2/3	Exa-cel (CASGEVY)	BCL11A erythroid enhancer	1	12 to 35	Vertex Pharmaceuticals	US, Belgium, Canada, France, Germany, Italy, UK	CD34^+^ HSPCs	CRISPR-Cas9	Completed	27 November 2018	[19]
SCD	NCT05329649	3	Exa-cel (CASGEVY)	BCL11A erythroid enhancer	1	2 to 11	Vertex Pharmaceuticals	US, Germany, Italy, UK	CD34^+^ HSPCs	CRISPR-Cas9	Recruiting	2 May 2022	No
BT	NCT05356195	3	Exa-cel (CASGEVY)	BCL11A erythroid enhancer	1	2 to 11	Vertex Pharmaceuticals	US, Canada, Italy, Germany, UK	CD34^+^ HSPCs	CRISPR-Cas9	Recruiting	3 May 2022	No
SCD & BT	NCT05477563	3	Exa-cel (CASGEVY)	BCL11A erythroid enhancer	1	12 to 35	Vertex Pharmaceuticals	US, Germany, Italy, Saudi Arabia	CD34^+^ HSPCs	CRISPR-Cas9	Recruiting	2 August 2022	No
SCD	NCT05951205	3	Exa-cel (CASGEVY)	BCL11A erythroid enhancer	1	12 to 35	Vertex Pharmaceuticals	n.d.	CD34^+^ HSPCs	CRISPR-Cas9	Not yet recruiting	31 July 2027	No
SCD & BT	NCT06647979	1	NA	BCL11A erythroid enhancer	1	13 to 40	Daniel Bauer	US	CD34^+^ HSCs	CRISPR-Cas9	Recruiting	1 December 2025	No
SCD	NCT05456880	1/2	Risto-cel (BEAM-101)	HBG1/HBG2 promoters	2	12 to 35	Beam Therapeutics	US	CD34^+^ stem cells	BE	Active, not recruiting	30 August 2022	[21]
BT	NCT05442346	NA	BRL-103	γ-globin	2	3 to 35	BRL Medicine	China	CD34^+^ HSPCs	G-BE	Suspended	25 December 2023	No
BT	NCT06024876	1	CS-101	HBG1/HBG2 promoters	2	6 to 35	CorrectSequence Therapeutics	China	CD34^+^ HSCs	BE	Active, not recruiting	26 August 2023	[22]
BT	NCT06065189	1	CS-101	HBG1/HBG2 promoters	2	3 to 17	Children’s Hospital of Fudan University	China	CD34^+^ HSCs	BE	Active, not recruiting	7 November 2023	No
BT	NCT06291961	1	CS-101	HBG1/HBG2 promoters	2	12 to 35	CorrectSequence Therapeutics	China	CD34^+^ HSCs	BE	Recruiting	18 April 2024	[22]
BT	NCT06328764	1	CS-101	HBG1/HBG2 promoters	2	6 to 35	CorrectSequence Therapeutics	China	CD34^+^ HSCs	BE	Enrolling by invitation	19 March 2024	[23]
SCD	NCT06565026	1	CS-101	HBG1/HBG2 promoters	2	12 to 35	CorrectSequence Therapeutics	China	CD34^+^ HSCs	BE	Recruiting	2 September 2024	[24]
SCD	NCT06506461	1	NA	HBG1/HBG2 promoters	2	18 to 24	St. Jude Children’s Research Hospital	US	CD34^+^ HSCs	CRISPR-Cas9	Recruiting	21 March 2025	No
SCD	NCT07000318	1	CS-206	HBG promoter (BCL11A binding site)	2	2 to 18	Children’s Hospital of Fudan University	China	CD34^+^ HSCs	BE	Not yet recruiting	30 June 2025	No
SCD	NCT04443907	1	OTQ923	HBG1/HBG2 promoters	2	2 to 40	Novartis Pharmaceuticals	US	HSPCs	CRISPR-Cas9	Terminated	25 August 2020	[25]
BT	NCT05444894	1/2	reni-cel (EDIT-301)	HBG1/HBG2 promoters	2	18 to 35	Editas Medicine	US, Canada	CD34^+^ HSPCs	CRISPR-Cas12a	Active, not recruiting	29 April 2022	[26]
SCD	NCT04853576	1/2	reni-cel (EDIT-301)	HBG1/HBG2 promoters	2	12 to 50	Editas Medicine	US, Canada	CD34^+^ HSPCs	CRISPR-Cas12a	Active, not recruiting	4 May 2021	[27]
BT	ChiCTR2100052858	0	RM-001	HBG1/HBG2 promoters	2	6 to 35	The 923rd Hospital of the People’s Liberation Army	China	CD34^+^ stem cells	CRISPR-Cas9	Recruiting	6 November 2021 *	[28]
BT	ChiCTR2100053406	0	RM-001	HBG1/HBG2 promoters	2	6 to 35	The First Affiliated Hospital of Guangxi Medical University	China	CD34^+^ stem cells	CRISPR-Cas9	Recruiting	20 November 2021 *	[28]
BT	NCT06041620	NA	VGB-Ex01	HBG1/HBG2 promoters	2	3 to 35	IHBDS Hospital, China	China	hematopoietic stem cells	CRISPR-Cas12b	Recruiting	31 August 2023	No
SCD	NCT04774536	1/2	CRISPR_SCD001	HBB promoter	3	12 to 35	Mark Walters, MD	US	CD34^+^ HSPCs	CRISPR-Cas9	Recruiting	18 September 2024	No
BT	NCT03728322	1	NA	HBB	3	2 to 60	Allife Medical	n.d.	iPSC-derived iHSCs	CRISPR-Cas9	Unknown	January 2019	No
SCD	NCT04819841	1/2	nula-cel	HBB	3	12 to 40	Kamau Therapeutics	US	CD34^+^ stem cells	CRISPR-Cas9	Recruiting	15 November 2021	[29]
HbH-CS	NCT06107400	1	RM-004	HBA2	3	12 to 35	The 923rd Hospital of the People’s Liberation Army	China	HSCs	C-BE	Recruiting	8 October 2023	[30]
HIV-1	NCT02500849	1	SB-728mR-HSPC	CCR5	3	18 to 75	City of Hope Medical Center	US	CD34^+^ HSPCs	ZFN	Active, not recruiting	10 March 2016	No
X-CGD	NCT06325709	1/2	NA	CYBB	3	18 to 75	NIAID	US	CD34^+^ HSPCs	BE	Recruiting	17 April 2024	No
P47-CGD	NCT06559176	1/2	PM359	NCF1	3	≥6	Prime Medicine	US, Canada, UK	CD34^+^ HSPCs	PE	Active, not recruiting	17 October 2024	[31]
X-SCID	NCT06851767	1/2	BE-HSPC-IL2RG	IL2RG	3	3 to 99	NIAID	US	HSPCs	BE	Enrolling by invitation	17 October 2024	No
XHIGM	NCT06959771	1/2	NA	CD40LG	3	≥37	NIAID	US	Combined HSPC and T-cell product	BE	Recruiting	16 July 2025	No
PKD	ChiCTR2300073795	1	NA	PKLR	3	6 to 17	Shanghai Children’s Medical Center	China	CD34^+^ stem cells	CRISPR	Recruiting	20 July 2023	No
LGMD	NCT05588401	1/2	GenPHSats	n.d.	n.d.	≥14	Simone Spuler, MD, Charite University, Berlin, Germany	n.d.	Primary human satellite cell-derived stem cells	CRISPR-Cas9	Unknown	1 July 2024	No

Disease abbreviations: BE: Base editor; BT: β-Thalassemia; C-BE: Cytosine base editor; G-BE: Glycosylase base editor; HbH-CS: HbH-constant spring (a severe form of alpha-thalassemia); IHBDS: Institute of Hematology and Blood Diseases; LGMD: Limb–girdle muscular dystrophy; MOA: Mechanism of action (see Table 1); NA: Not applicable (in the case of candidate names, no name is given); NIAID: National Institute of Allergy and Infectious Diseases; P47-CGD: p47phox autosomal recessive chronic granulomatous disease; PE: Prime editor; n.d.: Not disclosed; PKD: Pyruvate kinase deficiency; SCD: Sickle cell disease; X-CGD: X-linked chronic granulomatous disease; XHIGM: X-linked hyper IgM; X-SCID: X-linked severe combined immunodeficiency; ZFN: Zinc finger nuclease. * Date of registration: No start date available.

## Data Availability

No new data were created or analyzed in this study. Data sharing is not applicable to this article.

## Abbreviations

The following abbreviations are used in this manuscript:

A	Adenine
AAV6	Adeno-associated virus 6
ABEs	Adenine base editors
ANZCTR	Australia and New Zealand Clinical Trials Registry
BCL11A	B-cell lymphoma/leukemia 11A
BEs	Base editors
BT	β-thalassemia
C	Cytosine
Cas	Clustered regularly interspaced short palindromic repeats (CRISPR)-associated
CBEs	Cytosine base editors
ChiCTR	Chinese Clinical Trial Registry
CMN	CRISPR Medicine News
CRISPR	Clustered regularly interspaced short palindromic repeats
DSB	Double-stranded break
G	Guanine
gRNA	guide RNA
HCT	Hematopoietic cell transplant
HDR	Homology-directed repair
HPFH	Hereditary persistence of fetal hemoglobin
HSC	Hematopoietic stem cell
HSPC	Hematopoietic stem and progenitor cells
IHBDS	Institute of Hematology and Blood Diseases
indels	Insertions or deletions
LGMD	Limb–girdle muscular dystrophy
nCas9	Cas9 nickase
NHEJ	Non-homologous end joining
NIAID	National Institute of Allergy and Infectious Diseases
NIH	National Institutes of Health
PAM	Protospacer adjacent motif
pegRNA	prime-editing guide RNA
PKD	Pyruvate kinase deficiency
RT	Reverse transcriptase
SCD	Sickle cell disease
SCID	Severe combined immunodeficiency
T	Thymine
TDT	Transfusion-dependent β-thalassemia
X-CGD	X-linked chronic granulomatous disease
X-SCID	X-linked severe combined immunodeficiency
ZFN	Zinc-finger nuclease
